# Limb remote ischemic postconditioning protects cerebral ischemia from injury associated with expression of HIF-1α in rats

**DOI:** 10.1186/s12868-015-0235-6

**Published:** 2015-12-29

**Authors:** Yonghua Zong, Ling Jiang, Mingxiao Zhang, Fangfang Zhou, Wenqian Qi, Shuai Li, Huijun Yang, Yu Zou, Qingjie Xia, Xue Zhou, Xiaosong Hu, Tinghua Wang

**Affiliations:** Department of Morphology Lab and Department of Graduate, Chengdu Medical College, Sichuan, 610500 China; Institute of Neuroscience, Kunming Medical University, Kunming, 650031 China; Department of Anesthesiology and Institute of Neurological Disease, State Key Laboratory of Biotherapy, West China Hospital, Sichuan, 610041 China; Department of Histology, Embryology and Neurobiology, West China School of Preclinical and Forensic Medicine, Sichuan University, Chengdu, 610041 Sichuan China

**Keywords:** Cerebral ischemia–reperfusion, LRIP, MCAO, HIF-1α

## Abstract

**Background:**

Limb remote ischemic postconditioning (LRIP) can ameliorate cerebral ischemia–reperfusion injury (IRI), while the underlying mechanism remains elusive. Hypoxia-inducible factor 1α (HIF-1α) is an important transcription factor during cerebral ischemia damage. However, whether the neuroprotective effect of LRIP could be associated with HIF-1α is somewhat unclear. Here we tested the hypothesis that Limb remote ischemic postconditioning (LRIP) protecting brain from injury in middle cerebral artery occlusion (MCAO) rat model was associated with HIF-1α expression.

**Results:**

LRIP was conducted with 3 cycles of 10 min occlusion/10 min reperfusion at the beginning of reperfusion. The analysis of neurobehavioral function and triphenyltetrazolium chloride (TTC) staining showed the neurological deficit, brain infarct and cerebral edema, caused by ischemia–reperfusion injury (IRI), were dramatically ameliorated in LRIP administrated animals. Meanwhile, the result of Q-PCR and western blot revealed that the overexpression of HIF-1α induced by IRI could be notably suppressed by LRIP treatment.

**Conclusions:**

LRIP exhibits a protective effect against cerebral ischemia/reperfusion and the possible mechanism is associated with the suppression of HIF-1α in stroke rats.

## Background

Cerebral ischemia, the most common acute cerebrovascular disease, has been recognized as the third leading cause of mortality and disability in the worldwide. Up to date, few effective therapies could be used for the treatment of cerebral ischemia. Therefore, it is necessary to develop new methods for the treatment of brain ischemia and the involved mechanism should be investigated.

LRIP is designated as an emerging and developed postconditioning procedure, induced by repeated occlusion/reperfusion cycles in the bilateral femoral arteries. Previous studies have demonstrated that LRIP could effectively improve outcome in ischemic animal models [1–3], therefore possessing potential neuroprotection in alleviating brain edema, reducing cerebral infarct volume and blood–brain barrier disruption [4–6]. As a result, LRIP could be considered as an effective therapy for brain ischemia in future clinic practice. However, the potential protective mechanisms of LRIP are waiting to be clarified. HIF-1α, a primary component of the cellular and systemic response to hypoxia in mammals, consists of an oxygen-regulated α-subunit and a constitutive β-subunit [7]. As a master transcriptional regulator of numerous genes under hypoxic conditions, HIF-1α not only participates in regulating angiogenesis, glucose metabolism and cell survival during hypoxia [8–10], but also plays a crucial role in the development of apoptosis, inflammation activity [11–14]. Although HIF-1α has been widely known to be upregulated in rats subjected to cerebral ischemia, but the exact role of HIF-1α is still being debated. Moreover, no evidence is to address the role of HIF-1α in brain ischemia rats subjected to LRIP. In this study, we examined the neuroprotective effect of LRIP using MCAO rat model, indicated by neurological deficit score, infarct volume and brain edema. Then, using the analysis of Q-PCR, western blot and immunohistochemistry, we detected whether HIF-1α was involved in ischemic brain subjected to LRIP.

## Methods

### Animals and grouping

All SPF grade Sprague–Dawley rats weighing 250–280 g, were purchased from animal center of Sichuan University and the experimental protocol was approved by the Institutional Animal Care and Use Committee at Chengdu Medical College. At room temperature (22–25 °C), all rats were housed in a plastic cages (2 rats per cage) with 12-h light/dark cycle and had free access to food and water. All rats were randomly divided into three groups: sham, I/R and LRIP groups. The rats in sham group were subjected to surgical operation without occlusion of the right middle cerebral artery and LRIP treatment. The rats in I/R group were only suffered MCAO injury without LRIP treatment. The rats in LRIP group were subjected to MCAO group with LRIP treatment. Based on the reperfusion time, I/R and LRIP groups were respectively divided into following subgroups, including reperfusion 1, 3 and 7 days groups.

### Focal cerebral ischemia/reperfusion

Transient MCAO, as previously described [15], was used to induce focal cerebral ischemia. Rats were anesthetized deeply by injection of 3.6 % chloral hydrate (1 ml/100 g, IP). Heating lamps were used to maintain temperature at 36–37.5 °C. To perform brain ischemia, a midline neck incision in the neck was prepared. Then, the right common carotid artery (CCA), internal carotid artery (ICA) and external carotid artery (ECA) were exposed and isolated surgically. After the CCA was ligated close to its origin with a 3-0 silk suture, a type of 4.0 monofilament nylon suture with a slightly enlarged and rounded tip (Doccol Cooperation, Redlands, CA, USA) was inserted into the ICA,and advanced 18–20 mm until mild resistance, which indicated the middle cerebral artery (MCA) was effectively occluded. After 60 min of MCAO, the monofilament nylon suture was removed and MCA perfusion was restored. The sham group underwent the same procedure, but without occlusion of the right MCA. In limb remote ischemic postconditioning (LRIP) group, LRIP was conducted at the beginning of MCAO reperfusion. Briefly, the proximal hind limbs of each rat were tightened with rubber bands for 10 min as tight as possible, and then the rubber bands were loosened for 10 min. This process was repeated 3 cycles. The blood flow was completely interdicted and was confirmed by hypothermia, cyanosis and expansion in the hind limbs. When the procedures were finished, the animals returned to their cages and again allowed free access to food and water.

### Assessment of neurological function

The neurological deficit score (NDS) was performed with double-blind fashion at 24 h after IRI injury. To assure the brain damage in a similar degree as much as possible, the 1–2 scores animals according to the methods of Longa scoring [16] were included in the following experimental procedure. The method of Longa scoring is listed as follows: 0, no deficit; 1, failure to extend left forepaw fully; 2, circling to the left; 3, falling to the left; and 4, no spontaneous walking with a depressed level of consciousness.

The final neurobehavioral scoring in this study was performed according to Garcia JH [15], which contains six aspects: spontaneous activity (in case for 5 min); symmetry of movements (four limbs); symmetry of forelimbs (outstretching while held by tail); climbing wall of wire cage; reaction to touch on either side of trunk; response to vibrissae touch. Each aspects contain 0–3 points. The total scores range 3 from 18. The severity worsens as scores decreased.

### Measurement of infarct volumes and cerebral edema

The infarct volume was measured by triphenyltetrazolium chloride (TTC; Sigma) staining as described previously [17]. Three days after reperfusion, the brains were removed and cut into 4-mm-thick coronal sections, then the slices were incubated in 0.2 % solution of TTC diluted in 0.1 M sodium phosphate buffered saline (PBS) (pH 7.4) at 37 °C for 30 min and immersed overnight in 10 % formalin. The white color represented infarct tissue and red was normal tissue. The sections were photographed using a digital camera (Olympus, Japan). The infarct zone was demarcated and analyzed with Image J software (IPP, version 6.0). The ratio of infarct to normal volume was calculated as follows: [(area of the cortex in the non-ischemic hemisphere—area of the normal cortex in the ischemic hemisphere)/area of the non-ischemic cortex] × 100 % [2, 18]. Cerebral edema was counted from sections stained by TTC according to the formula: [(ipsilateral volume − contralateral volume)/contralateral volume] × 100 % [19].

### Quantitative RT-PCR analysis

According to the manufacturer’s instructions, the total RNA of infarcted area was isolated by Trizol reagent (Invitrogen, USA), and then was transcripted to cDNA reversedly with the RevertAidTM First Strand cDNA Synthesis kit (TaKaRa Biotechnology, Dalian, China). Quantitative RT-PCR analysis for the level of HIF-1α mRNA was performed by using Prime Script RT-PCR kits (Takara) as described. The mRNA level of β-actin was as an internal control. The sequences of primers used were shown as following: (forward) 5′-ACCCTCTGATTTAGCATGTAG-3′ and (reverse) 5′-GTAGGTTTCTGCTGCCTTGT-3′) for HIF-1α; (forward) 5′-GAAGATCAAGATCATTGCTCCT-3′ and (reverse) 5′-TACTCCTGCTTGCTGATCCA-3′) for β-actin. The real-time PCR program steps were 95 °C for 1 min, 45 cycles of 95 °C for 5 s, 60 °C for 5 s, and 72 °C for 20 s.

### Western blot

Brain tissues from infarct cortex were lysed and homogenized in RIPA lysis buffer (Beyotime, Jiangsu, China) supplied with 2 % of cocktail pill (Roche). Then suspension was centrifuged at 12,000 r/min for 10 min. And protein concentration was qualified with the BCA assay kit (Beyotime). 80 ug total protein was separated in 15 % SDS–polyacrylamide gel (SDS-PAGE) at 80 V for 2 h and transferred to PVDF membranes at 350 mA for another 2 h as well as was blocked with 5 % nonfat milk dissolved by TBST for 1 h at room temperature. Then, the blocked membranes were incubated overnight at 4 °C with the primary antibody of HIF-1α (rabbit, Abcam, 1:1000). After incubated with the primary antibody, the membranes were rinsed with TBST and incubated with the secondary antibody for 2 h (goat anti-rabbit IgG, ZSGB-BIO, China, 1:5000). Finally, ECL was added to membranes to develop image, which was conducted in Alpha Innotech (Bio-Rad).

### Immunohistochemistry and immunofluorescence

For immunohistochemistry, brains tissues from each group were harvested, post-fixed with 4 % paraformaldehyde and embedded in paraffin. Then, coronal sections (10 µm) were achieved at the level of the anterior commissure. When paraffin was removed, sections were incubated in 0.3 % H_2_O_2_ for 10 min. Then the sections were disposed with high pressure antigen retrieval for 2 min and blocked with 5 % goat serum at 37 °C for 20 min. Subsequently, they were incubated in the mixture of anti-HIF-1α antibody (rabbit, Abcam, 1:50) overnight at 4 °C. After washing in PBS, the sections were incubated with corresponding secondary antibody at 37 °C for 40 min. Finally, the antibody was visualized using DAB staining, then counterstained with hematoxylin, dehydrated, mounted, observed with a light microscope (Olympus/BX51, Tokyo, Japan) at 400× magnification. To compare the positive cell number, six fields from per section were captured and analyzed.

For immunofluorescence, sections were pretreated with previously, then incubated in the mixture of anti- HIF-1α antibody/anti-GFAP(mouse, 1:200, Proteintech), anti-HIF-1α antibody (rabbit, Abcam, 1:50)/anti-NeuN (mouse, 1:200, Abcam), After washed with PBS, they were incubated with fluorescence secondary antibody IgG (anti-mouse Alexa Fluor 488-conjugated, green, 1:100, Proteintech)/IgG (anti-rabbit Alexa Fluor 594-conjugated, red, 1:100, Proteintech) for 2 h at 37 °C. Then, the sections were rinsed with PBS and mounted by DAPI. Finally, the pictures were captured by fluorescence microscope (Olympus/BX51, Tokyo, Japan) at 400× magnification.

### Statistical analysis

All data expressed as the mean ± standard error of the mean (S.E.M.). Differences in quantitative data obtained from cerebral infarct area, brain edema, NDS, integrated densities of protein bands and immunohistochemical, the expression of genes were evaluated by one-way analysis of variance (ANOVA) and Student’s t test, p < 0.05 was considered as statistical significance. Statistical analysis was performed with SPSS version 17.0 software (SPSS Corp, USA).

## Results

### Effect of LRIP improving neurological function

**Fig. 1:**
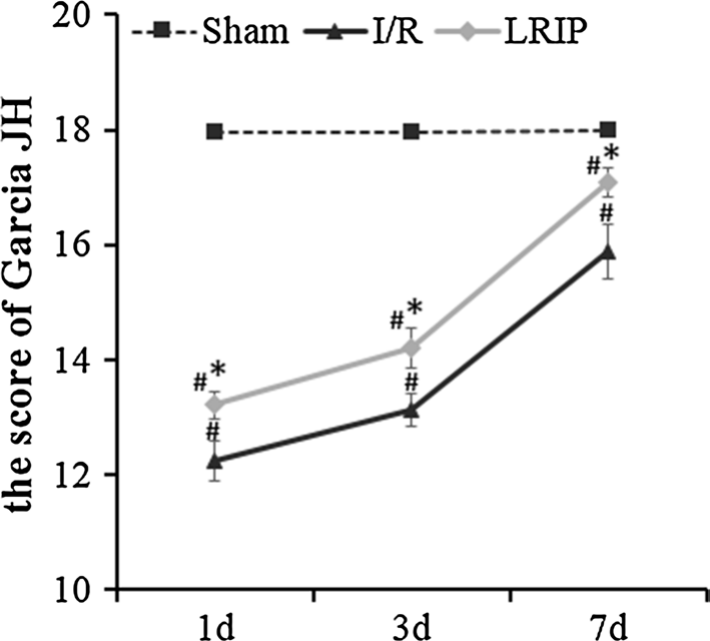
The analysis of Garcia JH scores. Quantitative evaluation of Garcia scores in different groups at day 1, 3 and 7. Compared with in sham group, the Garcia scores were obviously lower in I/R and LRIP group at day 1, 3 and 7 (^#^p < 0.05). Whereas, the LRIP administration could ameliorate the neurological function, indicated by increase of scores in LRIP group more than I/R group (*p < 0.05). The data is presented as mean ± SEM from eight animals per group

As shown in Fig. 1 (each group: n = 8), Garcia scores at different time in sham group were about 18, which indicated that there was no neurological deficit. Comparatively, after undergoing cerebral ischemia, the rats presented severity neurological deficit. At each time point of reperfusion, the Garcia scores in I/R group were lower than sham group (p < 0.05, Fig. 1). Moreover, the scores were notably increased compared with I/R group but it was still lower than sham group, when treated with LRIP (p < 0.05, Fig. 1). These confirmed that cerebral ischemia injury had resulted in neurological dysfunction, while LRIP treatment could inhibit this injured progress. These findings showed that the postconditioning treatment could improve the neurological function caused by MCAO injury.

### LRIP reduced cerebral infarct volume and suppressed cerebral edema

**Fig. 2:**
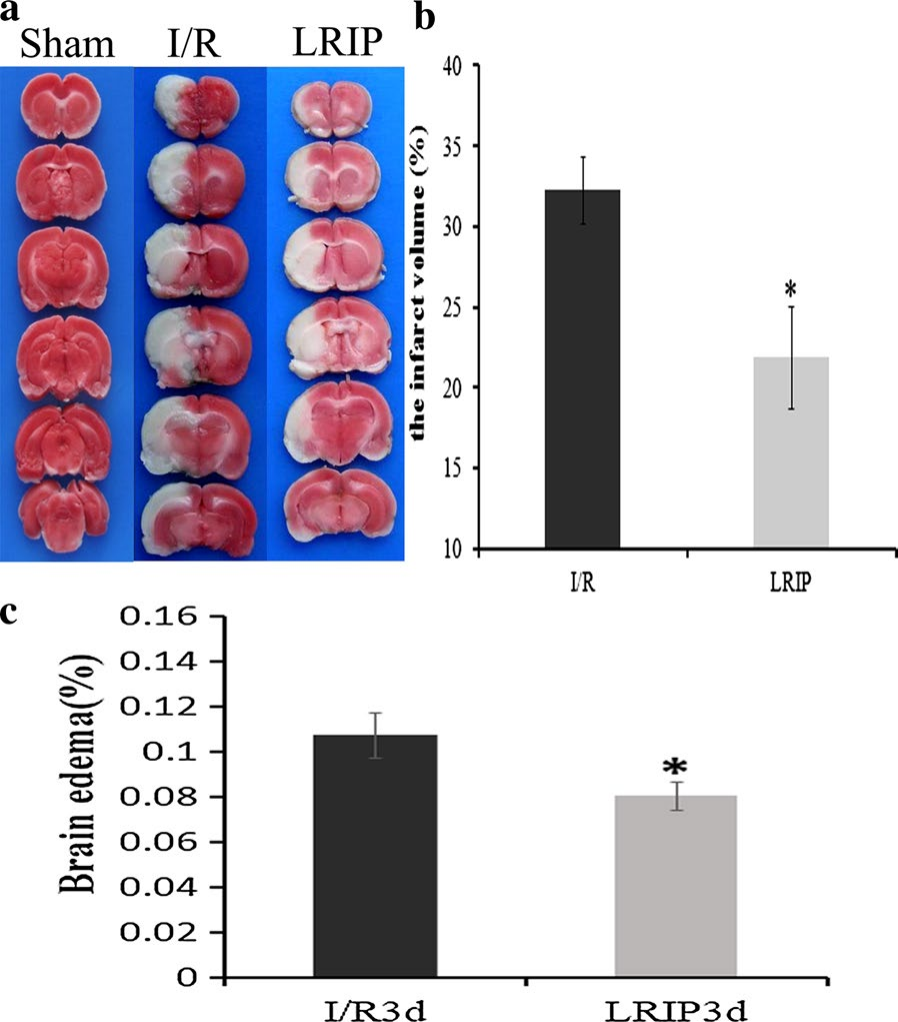
Measurement of infarct volume and edema formation at 3 days reperfusion after MCAO injury. **a** At 3 days after ischemia/reperfusion injury, TTC staining showed the brain infarct in I/R and LRIP group exhibited white color. **b** Quantitative evaluation of infarct volumes (%) in different groups was conducted at 3 days after ischemia/reperfusion injury. Compared with I/R group, the infarct volume was markedly diminished in LRIP administration group (*p < 0.05). **c** The brain edema (%) at 3 days was significantly reduced in LRIP administration group compared with I/R group. The data is presented as mean ± SEM from eight animals per group

To investigate whether LRIP could reduce infarct volume and cerebral edema in ischemic brain, we measured infarct volume and brain edema with TTC staining at 3d reperfusion (Fig. 2a). White zone in brain tissues represented damaged brain areas resulted from ischemia, and red zone represented normal brain tissues. Rats in sham group had no ischemic damage, showing red staining (Fig. 2a). I/R group exhibited that cerebral infarct volume was about 32.20 ± 2.16 %, whereas the LRIP decreased the white zone to 21.87 ± 1.98 % (p < 0.05; n = 5; Fig. 2b).Therefore, brain edema (%) was significantly reduced in LRIP group compared with I/R group. These result confirmed that LRIP treatment significantly decreased infarct volume and brain edema during cerebral ischemia injury.

### The activation and localization of HIF-1α

**Fig. 3:**
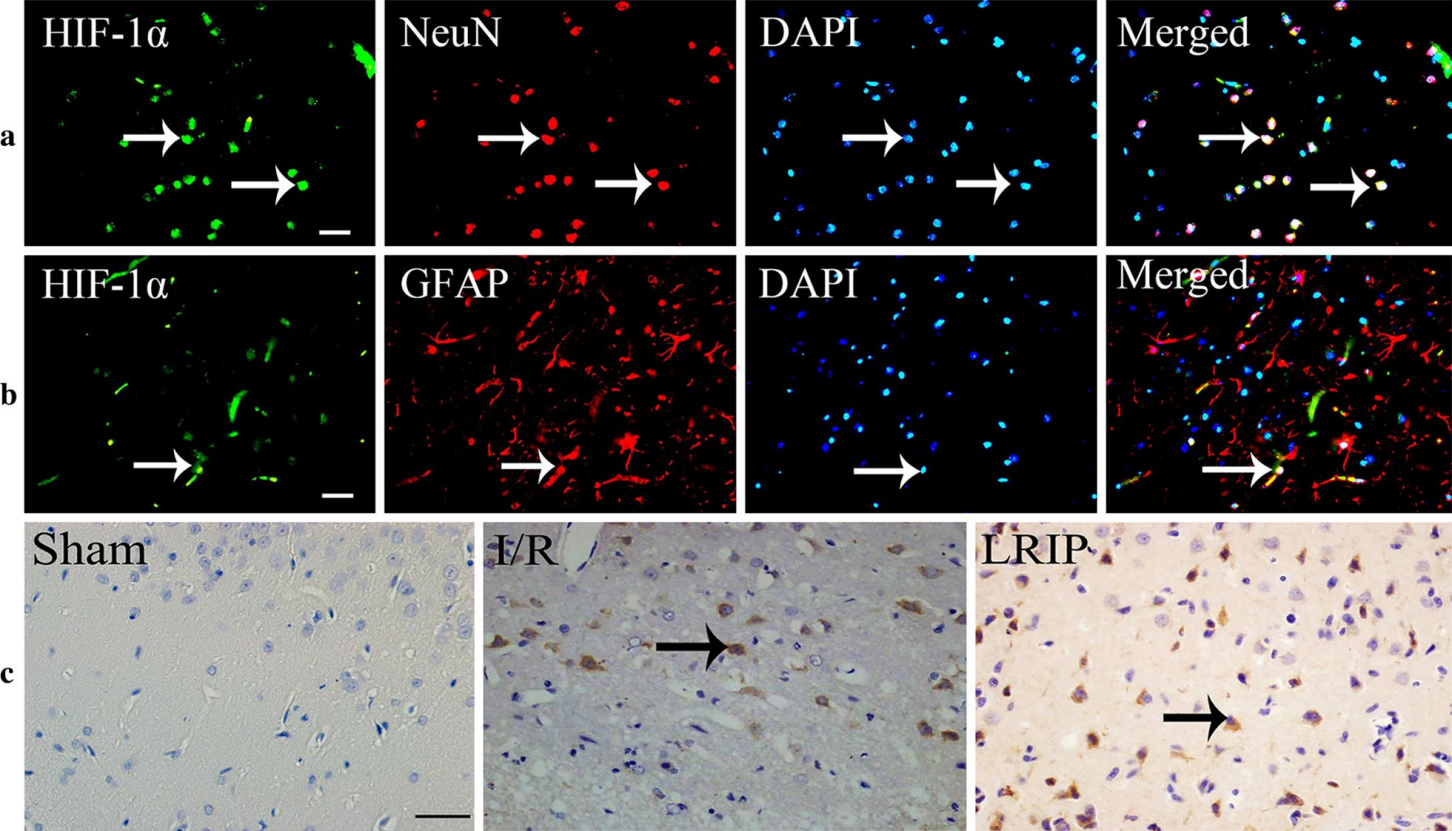
Immunohistochemical staining for HIF-1α in ischemic cerebral cortex at 3d reperfusion after MCAO injury. **a** Immunofluorescence double staining for HIF-1α/NeuN in neurons treated by LRIP. **b** Immunofluorescence double staining for HIF-1α/GFAP in astrocytes treated by LRIP. The immunofluorescence showed nucleus were stained by DAPI with *blue color* and HIF-1α were stained with* green color*, neurons were stained by NeuN and astrocytes were stained by GFAP with *red color*, and the co-expression of NeuN with HIF-1α within the same cell showed *blue color* (merged), the co-expression of GFAP with HFI-1α within the same cell showed *white color* (merged). The *arrow* showed double staining for HIF-1α/NeuN or HIF-1α/GFAP (*scale bar* = 20 μm). **c** HIF-1α immunoreactivity was marked with *brown color* in cytoplasm (*scale bar* = 20 μm)

**Fig. 4:**
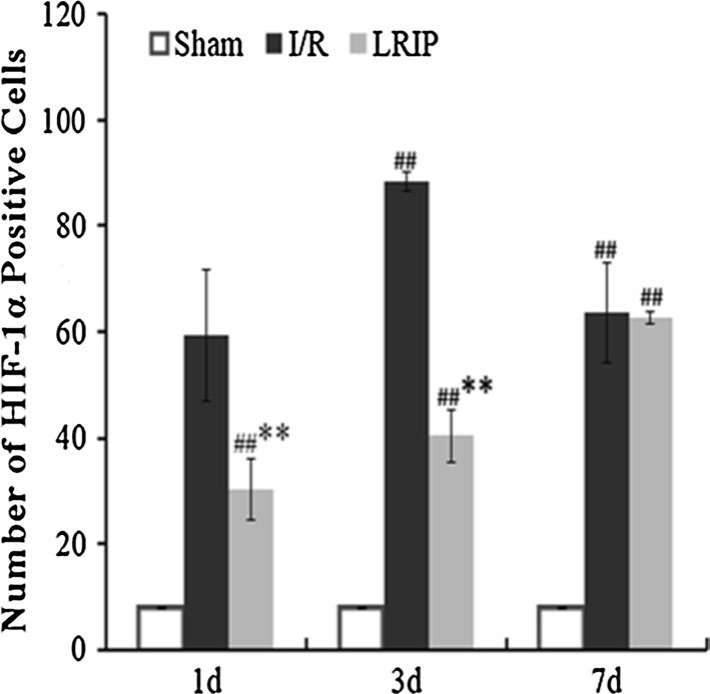
Quantitative analysis of HIF-1α positive cells. The analysis of HIF-1α positive cells in cerebral cortex. Compared to sham group, the number of HIF-1α presented a substantial increase in I/R and LRIP group (^##^p < 0.01) at 3 and 7 days. Compared to I/R group, the positive cells were downregulated in LRIP group at 1 and 3 days (**p < 0.01)

In this study, we tested the activation and distribution of HIF-1α in cerebral cortex by using immunohistochemistry. As shown in Fig. 3, immunofluorescence confirmed that HIF-1α was mainly located in cytoplasm of neurons, but few in astrocytes, while ischemia injury could induce HIF-1α increase. In sham group, we showed that HIF-1α exhibited a basic expression in cortex of brain. After ischemia–reperfusion, the number of HIF-1α positive neurons was significantly increased at each time point (p < 0.05; Fig. 4). Moreover, LRIP treatment decreased effectively the number of HIF-1α positive neurons and exhibited statistic significance at 1 and 3 days (p < 0.01; Fig. 4). These showed that ischemic postconditioning could suppress the expression of HIF-1α in neurons under ischemia condition.

### Expression of HIF-1α mRNA

**Fig. 5:**
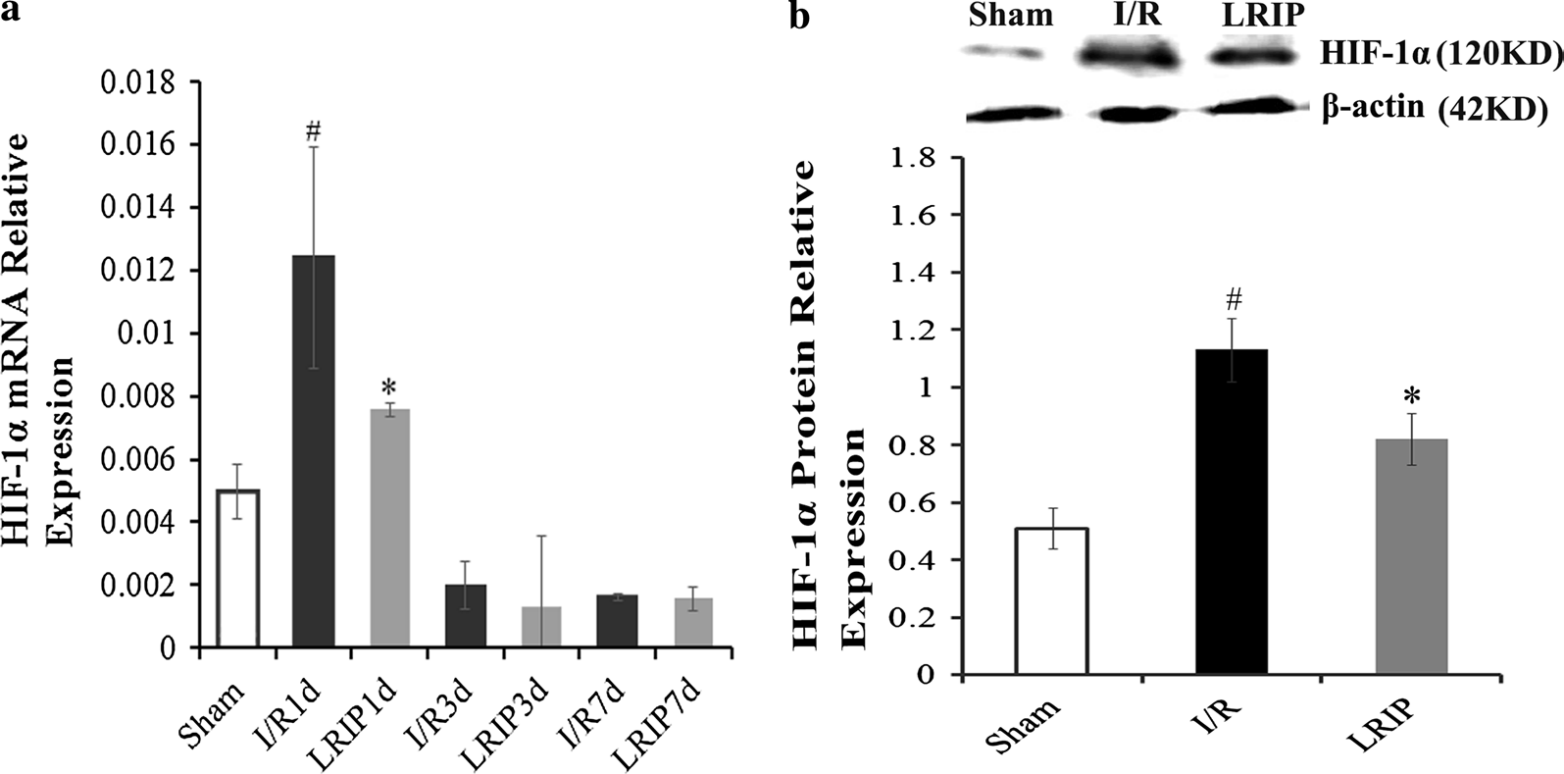
The mRNA expression of HIF-1α and the number of HIF-1α positive cells after reperfusion. **a** The expression of HIF-1α mRNA. The level of HIF-1α in I/R group was much higher than in sham group at 1 day post operation (^#^p < 0.05). But after LRIP administration, the level of HIF-1α was much lower than I/R group (*p < 0.05). At 3 days and 7 days, LRIP group still had a lower mRNA level of HIF-1α than I/R group, but the difference was not statistical significance. **b** The change of HIF-1α protein at 3 days. The *upper* one shows the western blot of β-action and HIF-1α. The *below* one is the quantitative analysis. LRIP suppressed the protein level of HIF-1α (^#^p < 0.05), which was upregulated by cerebral ischemia injury (*p < 0.05)

We determined the mRNA level of HIF-1α in the injured brain tissues at day 1, 3 and 7. Q-PCR showed that the expression of HIF-1α had a significant rising after ischemia and hypoxia (Fig. 5a). At day 1, the mRNA level of HIF-1α in I/R group was higher than seen in sham group (p < 0.05, Fig. 5a). However, LRIP treatment could partly reverse this alteration that mRNA expression of HIF-1α in LRIP group was significantly decreased at 1 day compared with in I/R group (p < 0.05). At 3 days and 7 days post operation, the expression of HIF-1α was gradually reduced in I/R group compared with sham group (p < 0.05), but there was no statistical change between I/R group and LRIP group (p > 0.05, Fig. 5a; n = 5).

### Level of HIF-1α protein

The protein expression of HIF-1α at 3 days was measured by western blot. As shown in Fig. 5b, the density of protein bands in sham, I/R and LRIP group was 0.51 ± 0.07, 1.13 ± 0.11, 0.82 ± 0.09, respectively. Compared with sham group, the expression of HIF-1α in I/R was increased significantly (p < 0.05, n = 5). However, in LRIP treated animals, the protein expression of HIF-1α was notably decreased (p < 0.05, n = 5). The result demonstrated that LRIP treatment could suppress the expression of HIF-1α increased by ischemia–reperfusion injury.

## Discussion

In this study, we found LRIP had the neuroprotective effect in brain ischemia rats, and the possible molecular mechanism is involving in the change of HIF-1α. Currently, focal ischemia induced by MCAO, which has been widely applied to research the effects and mechanisms of pre- and postconditioning on focal cerebral ischemia in the experiment animals. As a newly developed postconditioning procedure, LRIP was conducted immediately after focal cerebral ischemia–reperfusion establishment with three cycles of 10 min banding or loosing. As a result, an efficacious neuroprotection against brain ischemic injury was observed indicated by Garcia JH scores in LRIP administrated rats. TTC staining revealed that LRIP significantly reduced the cerebral infarct volume and relieved brain edema, which showed that LRIP could decrease cell death in ischemic area. Our observation added the fruitful evidence that was supported by previous reports [4, 20].

Possible mechanisms underlying the protective effect of LRIP may include amelioration of endothelial function, guarding the integrity of the blood brain barrier, modulation protein synthesis and nerve activity [2, 21], inhibition of apoptosis [22] and a decrease of reactive oxygen species (ROS) [5]. Recently, accumulating experiments focused on inflammatory interactions following brain ischemia, which were closely related to the pathogenesis of brain injury and might critically determine the outcome and prognosis of brain ischemia [23–25]. It has been reported that LRIP treatment could decrease expression of inflammation mediators interleukin-1beta (IL-1β) and IL-6, while they are important for neuroprotection under LRIP condition [26]. In our experimental model, LRIP exhibits significant neuroprotection in brain ischemia rats, and the molecular mechanism should involve the modulation of HIF-1α.

HIF-1α is a critical regulator contributing to adaptive responses to hypoxia, and HIF-1α can regulate cellular transductions under hypoxic milieu and inflammatory conditions [27–29]. During cerebral ischemic injury, HIF-1α was upregulated which may be involved in inflammation processes. Evidence showed that HIF-1α administration could regulate glycolytic capacity in myeloid cell to reduce myeloid infiltration to affect inflammatory progression. Suppression of HIF-1α in macrophages and neutrophils may result in inhibition of inflammatory reaction [30]. Whereas, HIF-1α deficiency may reduce the expression of tumor necrosis factor- alpha (TNF-α), interleukin-1 beta (IL-1β), cyclooxygenase-2, and inducible nitric oxide synthase levels in the ileal mucosa after trauma hemorrhagic shock (T/HS) [31], which might eventually contribute to structural and functional recovery. Literature showed that HIF-1α could regulate the expression of inducible nitric oxide synthase (iNOS) to affect inflammatory response during the ischemic phase of shock [32]. In addition, HIF-1α could significantly upregulate TNF-α to progress inflammatory response in the sepsis process formation and alveolar macrophages activated by lipopolysaccharide (LPS) [33]. By increasing the oxygen-dependent expression of T cell immunoglobulin and mucin domain protein (TIM)-3 in glial cells, HIF-1α could indirectly influence the infiltration of neutrophils into the hypoxic penumbra, which was regarded as a main cause of ischemic brain damage [34]. Therefore, HIF-1α is a crucial molecule in inflammatory response progress.

In our study, overexpression of HIF-1α was induced by IRI, while it was markedly attenuated in LRIP group during early cerebral ischemia. Therefore, the inhibition of HIF-1α may be critical mechanism for neuroprotection induced by LRIP. The present finding revealed LRIP exhibiting a neuroprotective role was related with the inhibition of HIF-1α, a critical inflammation mediator during brain ischemia. Additionally, inhibition of HIF-1α in LRIP may have other effects. As Liu BN [35] and Pichiule P [36] reported, HIF-1α could inhibit apoptosis pathway and improve angiogenesis in infarcted area via regulating vascular endothelial growth factor (VEGF), cleaving caspase-9 and erythropoietin (Epo). In our study, the expression of HIF-1α was markedly decreased in LRIP administration rats, which was associated with the improvement of neurological function, reduction of infarct size and brain edema. Therefore, we deduced that LRIP in MCAO rat model should be an effective method for the treatment of brain ischemia and the molecular events were possibly associated with the downregulation of HIF-1α expression.

## Conclusions

LRIP can significantly reduce cerebral damage caused by IRI, and the mechanisms are related to inhibiting the overexpression of HIF-1α to suppress inflammatory response. These available findings not only confirm the neuroprotective effect of LRIP, but also provide new information to understand the role of HIF-1α as a novel target for the treatment of brain ischemia in future clinic practice.

## Abbreviations

LRIP: Limb remote ischemic postconditioning
IRI: Ischemia-reperfusion injury
I/R: Ischemia reperfusion
HIF-1α: Hypoxia inducible transcription factors-1alpha
NDS: Neurological deficit score
MCAO: Middle cerebral artery occlusion
PBS: Sodium phosphate buffered saline
TTC: Triphenyltetrazolium chloride
TNF-α: Tumor necrosis factor-alpha
IL-1β: Interleukin-1beta
